# Using Mesoporous Silica-Based Dual Biomimetic Nano-Erythrocytes for an Improved Antitumor Effect

**DOI:** 10.3390/pharmaceutics15122785

**Published:** 2023-12-15

**Authors:** Ziyue Xi, Yingying Jiang, Zitong Ma, Qun Li, Xinran Xi, Chuanyong Fan, Shuang Zhu, Junjie Zhang, Lu Xu

**Affiliations:** School of Pharmacy, Shenyang Pharmaceutical University, Shenyang 110016, China; lnxiziyue@163.com (Z.X.); jiangyingying1016@163.com (Y.J.); mmmzt9992022@163.com (Z.M.); 17852250973@163.com (Q.L.); dzqhxxr@163.com (X.X.); fchuanyong@163.com (C.F.); zs2587009081@163.com (S.Z.); zhangjunjie256@163.com (J.Z.)

**Keywords:** biomimetic, erythrocyte-like nanoparticles, erythrocyte membrane, pH-sensitivity, antitumor

## Abstract

The nano-delivery system with a dual biomimetic effect can penetrate deeper in tumor microenvironments (TMEs) and release sufficient antitumor drugs, which has attracted much attention. In this study, we synthesized erythrocyte-like mesoporous silica nanoparticles (EMSNs) as the core loaded with doxorubicin (DOX) and coated them with calcium phosphate (CaP) and erythrocyte membrane (EM) to obtain DOX/EsPMs. The transmission electron microscopy (TEM), fluorescent co-localization and protein bands of SDS-PAGE were used to confirm the complete fabrication of EsPMs. The EsPMs with erythrocyte-like shape exhibited superior penetration ability in in vitro diffusion and tumor-sphere penetration experiments. Intracellular Ca^2+^ and ROS detection experiments showed that the CaP membranes of EsPMs with pH-sensitivity could provide Ca^2+^ continuously to induce reactive oxide species’ (ROS) generation in the TME. The EM as a perfect “camouflaged clothing” which could confuse macrophagocytes into prolonging blood circulation. Hemolysis and non-specific protein adsorption tests proved the desirable biocompatibility of EsPMs. An in vivo pharmacodynamics evaluation showed that the DOX/EsPMs group had a satisfactory tumor-inhibition effect. These advantages of the nano-erythrocytes suggest that by modifying the existing materials to construct a nano-delivery system, nanoparticles will achieve a biomimetic effect from both their structure and function with a facilitated and sufficient drug release profile, which is of great significance for antitumor therapy.

## 1. Introduction

In recent years, it has been a research hotspot to obtain inspiration from nature, imitate the structure and function of natural substances, and develop some materials from the perspective of bionics to achieve specific applications. In the field of nano-drug delivery involved in this study, biomimetic materials can be designed as new carriers for the targeted delivery and sustained release of drugs at tumor sites for improving antitumor effects. In the last decades, the obstacles of the tumor microenvironment (TME), including vascular extravasation, tumor interstitium obstruction, tumor site accumulation, and intracellular drug release restriction, limited the antitumor effect of nanomedicines [1,2]. Recently, it has been noticed that nanoparticles with a biomimetic shape could overcome a series of physiological obstacles in the TME [3,4]. There are a variety of material candidates that can be transformed based on shape bionics. Among them, the mesoporous silica nanoparticles (MSNs) have attracted increasing attention due to their high synthesis controllability and satisfying physicochemical properties such as their adjustable shape and size, easy surface functionalization, superior pore structure, large pore volume, and specific surface area [5,6,7]. Therefore, many biomimetic shapes of MSNs have been developed as worm-like [8], virus-like [9], and erythrocyte-like [10] shapes for delivering drugs efficiently. Especially, it has been shown that erythrocyte-like MSNs (EMSNs), which simulate the shape of erythrocytes, displayed higher accumulation at tumor sites and preferably longer penetration distances [11].

However, the naked EMSNs still suffer from poor biocompatibility and bursting or premature drug leakage. At present, coating nanoparticles with various membranes has been considered one of the most promising drug delivery strategies to improve biocompatibility, enhance cellular internalization, and optimize drug release profiles for cancer treatment [12,13,14,15,16]. As reported, calcium phosphate (CaP), which is able to be extracted from teeth and bones, has been noticed due to its preferable biodegradability [17] and pH-sensitivity [18]. In particular, calcium phosphate possesses an intelligently pH-responsive degradation behavior through generating Ca^2+^ at the TME. The accumulation of intracellular Ca^2+^ disrupts the osmotic pressure balance of lysosomal membranes and induces the generation of reactive oxygen species (ROS) to cause tumor cells’ necrosis [19,20,21,22]. Based on these strengths, CaP has great potential to be used as an intelligent pore gatekeeper for tumor therapy. For example, Liu et al. [23] synthesized a multifunctional MSN modified with poly(N-isopropylacrylamide)-co-acrylic acid and a calcium phosphate membrane (MSCNs) to transport DOX, which exhibited an excellent pH-responsive drug release and targeting ability. But CaP, as inorganic materials, is accompanied by a relatively low biocompatibility and high clearance rate due to the phagocytosis of the reticuloendothelial system [24], inducing a limited cellular internalization. In this way, endogenous substances, especially the erythrocyte membrane (EM), sheds light on this issue because of its superior biocompatible property in avoiding being identified and eliminated by macrophages [25,26], which could effectively address the dilemmas of inorganic materials. Firstly, there are ample CD47 marker proteins on EM, which can transmit signals to refrain from being phagocytized and cleared [27,28]. Furthermore, compared with organic radical polymers like polyethylene glycol (PEG) [29], poly [n-(2-hydroxypropyl) methacrylamide] (PHPMA) [30], and poly (L-glutamic acid) [31], EMs camouflaged on the surface of MSNs mitigate the production of anti-PEG-IgG and other substances to reduce the clearance rate [32]. Therefore, when coated with EM and CaP dual membranes, the nanoparticles may provide an exciting opportunity for not only improving biocompatibility, but also controlling the sustained release of drugs at tumor sites.

As mentioned above, whether it is based on the shape or the coated membrane to modify nano-drug-delivery carriers, the single biomimetic effect is limited and one-sided. More and more studies have begun to combine shape and membrane modification, focusing on the dual biomimetic effect of both structure and function [33,34]. For instance, Yu et al. [11] constructed a discoidal nano-drug-delivery system in which perfluorohexane (PFH) and doxorubicin (DOX) were encapsulated in discoidal MSNs decorated with EMs for mitigating the hypoxic environment to achieve a chemotherapy effect. Based on the dual biomimetic effect from the shape and membrane of erythrocytes, compared them with spherical nanoparticles, nano-erythrocytes exhibit a good performance in lessening the side effects and enhancing tumor penetration and movement, and nano-erythrocytes can escape from the phagocytosis of the immune system and have a longer circulation time.

**Scheme 1:**
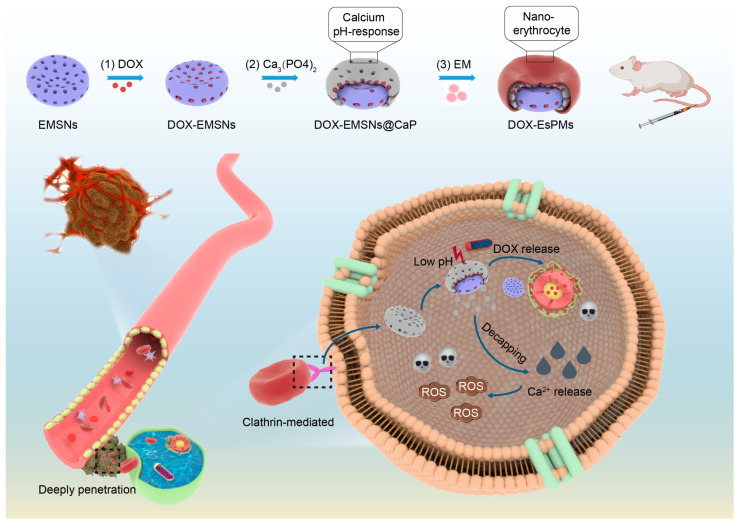
Schematic illustration of the synthesis procedure and antitumor mechanism of DOX-EsPMs.

In this work, we constructed dual biomimetic core–shell nano-erythrocytes with pH-responsive modifications in the TME, which could conquer the obstacles of the TME to facilitate nano-erythrocytes’ enrichment at tumor sites and sustained drug release for enhancing antitumor effects. As shown in Scheme 1, the core–shell structure of nano-erythrocytes (DOX/EsPMs) were composed of erythrocyte-like MSNs (DOX/EMSNs) and were coated with CaP and EM dual membranes. Due to DOX/EMSNs with erythrocyte-like shapes as their core, the DOX/EsPMs exhibited a deeper penetration when moving toward the surfaces of tumor cells and enhanced endocytosis than that of spherical MSNs. The CaP membranes of DOX/EsPMs, as pore blockers with high pH sensitivity, increased the drug accumulation at the tumor sites precisely. At the same time, the aggregation of Ca^2+^ via the degradation of CaP induced an explosive production of ROS, and then promoted the apoptosis of tumor cells. The EM of DOX/EsPMs reduced plasma protein adsorption, restrained immune phagocytosis and prolonged blood circulation time effectively. In conclusion, nano-erythrocytes with biomimetic effects of structure and function could achieve the precise release and enrichment of chemotherapy drugs at tumor sites, and provide a promising example for the construction of dual biomimetic nano-drug-delivery platforms.

## 2. Materials and Methods

### 2.1. Materials

Cetyltrimethylammonium bromide (CTAB) and sodium dihydrogen phosphate were purchased from Tianjing Kemiou Chemical Reagent Co., Ltd. (Tianjing, China). Mercaptoundecanoic acid (MUA) and doxorubicin hydrochloride (DOX) were purchased from Aladdin Chemistry Co., Ltd. (Shanghai, China). Tetraethoxysilane (TEOS) was purchased from Tianjin Damao Chemical Reagent Factory. Calcium chloride was purchased from Tianjin Hengxing Chemical Reagent Manufacturing Co., Ltd. (Tianjing, China). Dynasore, chlorpromazine and amiloride were purchased from Dalian Meilun Biotechnology Company (Dalian, China).

### 2.2. The Synthesis of Nanoparticles

Nano-erythrocytes were prepared according to the previously reported method with a slight adjustment [10]. Briefly, CTAB (1.10 mmoL) and MUA (0.46 mmoL) were dispersed in the mixed solvent of distilled water (120 mL) and ethanol (40 mL), then the mixture was sonicated to form a uniform micelle solution as surfactant template. Next, TEOS (2 mL) and ammonium hydroxide (1.86 mL) were added dropwise and stirred continuously for 3 h at 25 °C. The reaction mixture was centrifuged and washed three times with ethanol, then dried in a vacuum drying oven at 80 °C for 24 h. To remove the surfactant template, the crude product was calcined in a crucible with gradient temperature from 100 to 600 °C for 6 h. The acquired solid product as the core of nano-erythrocytes was called EMSNs.

EMSNs (10 mg) were dispersed in a component solvent composed of cyclohexane (7.5 mL), Triton X-100 (1.77 mL) and hexyl alcohol (1.6 mL) to form a suspension. After mixing evenly, calcium chloride solution (0.46 M, 250 μL) and sodium dihydrogen phosphate solution (2.92 M, 250 μL) were added dropwise and stirred for 10 h at 25 °C to form the CaP membrane. Finally, the sample named EMSNs@CaP was collected by using centrifuge with 10,000 rpm for 10 min. EsPMs were prepared by mixing EMSNs@CaP and erythrocyte membrane (EM) at a weight rate of 1.25:1, sonicating under ice bath for 3 min and centrifuging at 10,000 rpm for 10 min.

Spherical mesoporous silica (SMSNs) was prepared according to the following method. Triethanolamine (110 mg) and CTAB (0.4 g) were dispersed in distilled water (20 mL) and sonicated to obtain transparent solution. The solution was stirred in an oil bath (95 °C, 1 h) and then TEOS (1.5 mL) was added dropwise. After reacting for 1 h, precipitate was collected by centrifuging at 10,000 r/min for 10 min, washing three times with ethanol. The precipitate was dispersed in a mixed solution of ethanol (20 mL) and concentrated hydrochloric acid (1 mL), and stirred at 60 °C for 8 h by reflux to remove template. After that, the precipitate was washed with ethanol and deionized water three times, centrifuged and dried to collect SMSNs. Subsequently, SMSNs were coated with CaP membranes and EMs by the same method as above to obtain SsPMs.

### 2.3. The Characterization of Nanoparticles

The morphology of nanoparticles (EMSNs, EMSNs@CaP, EsPMs) and SsPMs were investigated by TEM (JSM-6510A, JEOL, Tokyo, Japan) at 200 kV. The size distribution and zeta potential were tested by using a Zetasizer NanoZS90 (Malvern Instruments Co., Ltd., Malvern, UK). The mesoporous structure of EMSNs and EMSNs@CaP was determined by SAXS scatter meter (Anton Paar GmbH, Graz, Austria). The detection conditions were a scanning range of 0~10° (2θ), scanning step size of 0.02°, and scanning speed of 0.6°/min. Nitrogen adsorption and desorption analysis was performed to characterize the pore structure of EMSNs and EMSNs@CaP by applying an SA3100 surface area and pore size analyzer (Beckman Couler, Brea, CA, USA). The co-localization of EsPMs labeled with FITC (core) and DiI (EM) was observed under confocal laser scanning microscope (Carl Zeiss AG, Oberkochen, Germany). The surface membrane proteins of nanoparticles coated with EMs were separated and detected by sodium dodecyl sulfate-polyacrylamide electrophoresis (SDS-PAGE), and the results were observed under the gel imaging system.

### 2.4. An In Vitro Ca^2+^ Release Study

The complex chromogenic reaction was applied to evaluate the Ca^2+^ release condition of CaP membrane coated on nanoparticles. Briefly, calcium carboxylic acid indicator (C_21_H_14_N_2_O_7_S, 400 μL), NaOH solution (4 M, 1.5 mL), and test solution were mixed completely and then the absorbance of the solution was determined by UV-vis spectrophotometer (756 PC, Shanghai, China) at 560 nm.

EMSNs@CaP, EsPMs and SsPMs were dispersed in PBS 7.4 to dilute the concentration of Ca^2+^ (3.58 mg/mL). Each dialysis bag was added to test solution (500 μL) and placed in a flask containing 20 mL PBS (pH 5.0 or 7.4). Then, flasks were shaken in a gas bath shaker (37 °C, 100 rpm). At predetermined time intervals, 2 mL dissolution medium was withdrawn as test sample and corresponding fresh medium was added to maintain a constant dissolution volume. The Ca^2+^ amount of the transferred dissolution medium was determined by the above complex chromogenic reaction. All the above experiments were carried out in triplicate.

### 2.5. Drug Loading Capacity

EMSNs (10 mg) and DOX (5 mg) were dispersed uniformly in 4 mL PBS 7.4 and stirred for 36 h at 25 °C in the dark. After reaction, the solution was centrifuged at 8000 rpm for 10 min and washed with PBS for three times. The supernatants after centrifugation were collected to determine the absorbance at 480 nm by UV-vis spectrophotometer and precipitates were dried to obtain drug-loaded EMSNs (DOX/EMSNs). The DOX/EMSNs@CaP and DOX/EsPMs were synthesized according to the “Synthesis of Nanoparticles” section with a slight change, which was that EMSNs were replaced with DOX/EMSNs, and all other operations remained unchanged. The construction method of DOX/SsPMs was the same as above. The drug loading (DL%) was calculated by the equation as follows:

(1) $$\mathrm{DL}\left(\%\right)=\frac{M_{1}-C\times V}{M_{1}+M_{2}}\times 100$$

where M_1_ was the weight of initial DOX, M_2_ was the weight of nanoparticles, C was the concentration of DOX in the supernatant, and V was the total volume of supernatant.

### 2.6. An In Vitro Drug-Release Study

The in vitro drug release behavior was investigated by a dialysis method. Every dialysis bag was added to 500 μL drug-loaded nanoparticle solution containing 0.6 mg DOX, then immersed in 100 mL PBS 5.0 or PBS 7.4 under continuous shaking (37 °C, 100 rpm). At the pre-designed time point (1 h, 2 h, 4 h, 6 h, 8 h, 12 h, 24 h, 36 h, 48 h, 72 h, 96 h, 120 h, 144 h), 5 mL of dissolution medium was taken out, and the same volume of corresponding fresh PBS was supplemented. The absorbance of the dissolution medium was measured at 480 nm by UV-vis spectrophotometer, and the released amount of DOX was calculated by standard curve equation. All the above experiments were carried out three times.

### 2.7. The Hemolysis Test and Non-Specific Protein Adsorption

Hemolysis test and non-specific protein adsorption experiment were conducted to assess the biocompatibility of nanoparticles. In short, samples (EMSNs, EMSNs@CaP, EsPMs) were dispersed in saline, and then mixed with 2% erythrocyte suspension (25, 50, 100, 200, 400, 600 μg/mL), and then incubated for 3 h at 37 °C. Moreover, 2% erythrocyte suspension mixed with saline or deionized water was regarded as negative control or positive control groups, respectively. Afterwards, the mixtures were centrifuged at 4000 rpm for 5 min, and the absorbances of supernatants were measured at 540 nm by microplate reader. The hemolysis rate was calculated by the equation:

(2) $$\mathrm{Hemolysis}\;\mathrm{Rate}\left(\%\right)=\frac{{\mathrm{OD}}_{S}-{\mathrm{OD}}_{N}}{{\mathrm{OD}}_{P}-{\mathrm{OD}}_{N}}\times 100$$

where OD_S_ was the absorbance of each sample, OD_N_ was the absorbance of the negative control, and OD_P_ was the absorbance of the positive control.

The UV-vis spectrophotometric method and SDS-PAGE analysis were applied to evaluate the non-specific protein adsorption of nanoparticles. EMSNs, EMSNs@CaP, EsPMs and SsPMs solutions were incubated with plasma to obtain mixed solution. The supernatants were determined at 562 nm by microplate reader. The precipitate was dispersed in PBS 7.4, added to RIPA lysis buffer, then boiled to conduct SDS-PAGE analysis. The protein absorption amounts were calculated according to the equation:

(3) $$q\left(\%\right)=\frac{\left(C_{0}-C_{X}\right)\times V}{m}\times 100$$

where C_0_ and C_X_ was the initial and remaining plasma protein concentration, V was the volume of the mixed solution, and m was the weight of each sample.

### 2.8. The Degradation Study

In vitro degradation condition of nanoparticles was evaluated by weight loss method. In brief, samples were weighed accurately and put into centrifuge tubes containing 5 mL PBS 7.4 or PBS 5.0 (1 mg/mL). Then, the solutions were shaken gently at 37 °C for 18 days and the test solutions were collected at 3, 6, 9, 12, 15 and 18 days. The collected medium was centrifuged at 10,000 rpm for 10 min, washed with deionized water three times, then the precipitate was dried and weighed to calculate the degradation rate by the following equation:

(4) $$W_{L}\left(\%\right)=\frac{W_{1}-W_{t}}{W_{1}-W_{0}}\times 100$$

where W_0_ was weight of the empty tube, W_1_ was total weight of the empty tube and EMSNs before the degradation experiment, and W_t_ was total weight of the sample and tube after drying.

### 2.9. Multiple Particle Tracking (MPT)

Multiple particle tracking (MPT) was applied to investigate the movement of nanoparticles in simulated tumor stroma. Hydroxyethyl cellulose was developed to simulate extracellular matrix (ECM), because its fibrous network structure and pore size distribution are similar to ECM in tumor tissue as revealed in the previous literature [35]. A total of 5 μL FITC-labeled nanoparticles (100 μg/mL) were dispersed in 100 μL hydroxyethyl cellulose solution, then vortexed and incubated at 37 °C for 20 min. The Brownian motion of nanoparticles was observed under a fluorescence microscope, then a video was shot for 10 s at an exposure rate of 32.6 ms per frame. Image J2 (Fiji) software was used to analyze video and process data. The mean squared displacement (MSD) and effective diffusivity (Deff) were calculated by the following equations:

(5) $${\mathrm{MSD}}_{t}={\;\left(x_{t}-x_{0}\right)}^{2}+{\left(y_{t}-y_{0}\right)}^{2}$$

(6) $$D_{\mathrm{eff}}={\mathrm{MSD}}_{t}/4t$$

where x and y were the coordinates of nanoparticles, and t was the time scale.

### 2.10. In Vitro Tumor Sphere Penetration

The penetration experiment of tumor spheres was used to further explore the penetration of nanoparticles at tumor sites. A total of 2% (*w*/*v*) agarose solution was sterilized using high pressure and high temperature, and then added to the cell culture well. After standing for 15 min, the agarose was completely solidified. The prepared 4T1 cells and DMEM medium were added to the well and placed in an incubator at condition of 5% CO_2_ at 37 °C for one week. Next, 200 μL RITC-labeled EMSNs, EMSNs@CaP, SsPMs and EsPMs were incubated with cultured tumor sphere for 4 h, rinsed, fixed with 4% paraformaldehyde, and observed under CLSM.

### 2.11. The Cell Cytotoxicity Study

The cytotoxicity of blank nanocarriers and DOX-loaded nanoparticles was evaluated by MTT assay. The 4T1 cells were cultured into 96-well plates at a density of 1 × 10^4^ cells/well and incubated for 12 h. Then, the medium was transferred and cells were treated with free DOX and DOX-loaded nanoparticles at serial DOX concentrations (6.25 μg/mL, 12.5 μg/mL, 25 μg/mL, 50 μg/mL, and 100 μg/mL) for 24 h. Afterwards, the culture medium was removed and 96-well plates were washed with PBS. MTT solution (0.5 mg/mL, 100 μL) was added to each well and incubated for another 4 h. Finally, MTT solution was removed, and each well was added 100 μL DMSO and shaken at 37 °C for 20 min to dissolve the formazan crystal. Additionally, nanocarriers at equivalent DOX concentration were also incubated with cells to investigate the cytotoxicity of carriers in the same method. The absorbance was measured at 490 nm using a microplate reader.

### 2.12. Cellular Uptake and Mechanism Studies

The 4T1 cells cultured in 12-well plates at a density of 3 × 10^5^ cells/well were selected to evaluate endocytosis efficiency of nanoparticles. DOX-loaded nanoparticles (EMSNs, EMSNs@CaP, EsPMs and SsPMs) were incubated with cells for 4 h and 8 h, respectively. After removing the medium, the 12-well plates were washed three times with PBS. Then, cells were fixed by paraformaldehyde, stained with DAPI, and finally observed using CLSM.

The 300 μL DOX-loaded nanoparticles were added to each well and incubated with 4T1 cells for 4 h and 8 h, respectively. After incubation, 300 μL RIPA lysis buffer was added to each well for 20 min and the mixed solution was centrifuged. The 200 μL supernatant was used to measure DOX fluorescence and other 20 μL supernatant was added into 200 μL BCA solution and incubated at 37 °C for 20~30 min. The absorbance was measured at 562 nm using a microplate reader, and the cellular uptake amount of different carriers was obtained.

Moreover, filipin (ELP), dynasore (DNS), and chlorpromazine (CPZ) were used as different kinds of cellular uptake inhibitors to explore potential mechanism of nanoparticles endocytosis. The 4T1 cells were treated with 500 μL CPZ (10 μg/mL), FLP (5 μg/mL) and DNS (20 μg/mL) for 2 h. After washing with PBS, FITC-labeled nanoparticles were added to the wells and incubated for 2 h, respectively. After PBS washing, paraformaldehyde fixation and DAPI staining, the cells were observed using CLSM. The quantitative data was obtained by BCA kit and the method was the same as cellular uptake quantification. All the experiments were conducted three times.

### 2.13. Mitochondrial Co-Localization

The 4T1 cells of each well were incubated with 200 μL of RITC-grafted nanoparticles (EMSNs, EMSNs@CaP, EsPMs and SsPMs) for 2 h. After removing the medium, PBS was used to wash it 3 times, and then 200 μL Mito Tracker was added and incubated for 30 min to label mitochondria. Next, the 4T1 cells were fixed with paraformaldehyde for 15 min, and then stained with 300 μL DAPI for 10 min. After washing with PBS, the 4T1 cells were observed and photographed under a CLSM.

### 2.14. Intracellular Ca^2+^ Release and ROS Detection Studies

The 500 μL nanoparticle solutions (EMSNs, EMSNs@CaP, SsPMs and EsPMs) were incubated with cells for 4 h. After removing the medium and washing with PBS, the cells of every well were mixed with 300 μL Fluo-4 AM (2 µM) for 30 min to label Ca^2+^, fixed by 4% paraformaldehyde, and then stained with DAPI to label the nucleus. The fluorescent images were detected using CLSM and the Ca^2+^ release amount was quantitively analyzed by BCA kit.

The intracellular ROS was tested using 2′,7′-dichlorofluorescein diacetate (DCFH-DA) as an index. The process of ROS detection was almost the same as the intracellular release of Ca^2+^. The difference was that 300 μL Fluo-4 AM (2 μM) was replaced by 300 μL DCFH-DA solutions (5 μM) for staining. The fluorescence intensity of ROS was observed under CLSM, and the ROS was quantitatively detected by BCA kit.

### 2.15. The In Vivo Circulation Study

The in vivo circulation capacity of nanoparticles was investigated by determining fluorescence intensity of orbital blood extracted from SD rats. SD rats were injected with FITC-labeled nanoparticles (EMSNs, EMSNs@CaP, EsPMs and SsPMs) via the tail vein in doses of 10 mg/kg. Blood samples were collected at predetermined time intervals (5 min, 15 min, 0.5 h, 1 h, 2 h, 4 h, 6 h, 8 h, 10 h, 12 h, 24 h, and 48 h) and then centrifuged for 10 min at 1500 rpm. The FITC fluorescence intensity of supernatant was measured at 488 nm/519 nm (excitation/emission wavelength) by microplate reader. The relative fluorescence signal (the fluorescence intensity of the supernatant at different time points to the fluorescence intensity of the supernatant at 5 min) was used to represent the in vivo circulation capacity of the nanoparticles.

### 2.16. In Vivo Pharmacodynamics and Biosafety Studies

The 4T1-tumor-bearing Balb/c mice model was built by subcutaneously injecting 4T1 cells solutions with 1 × 10^6^ cells per rat. Then, rats were randomly divided into six groups (*n* = 5) and injected with different solutions (Saline, DOX, DOX/EMSNs, DOX/EMSNs@CaP, DOX/EsPMs and DOX/SsPMs) at a 2 mg/kg equivalent dose of DOX through the tail vein, respectively. The body weight, tumor length (L), and tumor width (W) were recorded every other day for 14 days. The relative volume changes were evaluated via V/V0 (V0 was the initial tumor volume before treatment) for reflecting the antitumor effect. After 14 days treatment, tumor tissues and organs were separated, weighed precisely, fixed, dehydrated, and analyzed for H and E staining. Finally, the slices were observed by a microscope.

### 2.17. Statistical Analysis

All data were represented as mean ± SD. The statistical significance was evaluated by Student’s *t*-test (comparing two groups) and one-way ANOVA (comparing multiple groups). Statistical significance was set as *p* > 0.05, * *p* < 0.05, ** *p* < 0.01, *** *p* < 0.001, and **** *p* < 0.0001.

## 3. Results and Discussion

### 3.1. The Characterization of Nanoparticles

**Figure 1:**
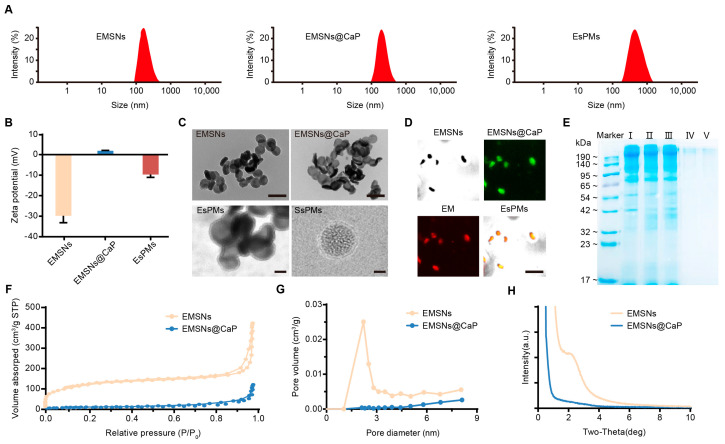
Characterization of nanoparticles. (**A**) Size distribution curves and (**B**) zeta potential of nanoparticles. (**C**) TEM images of EMSNs, EMSNs@CaP, scale bar, 200 nm; EsPMs and SsPMs, scale bar, 50 nm. (**D**) CLSM images of EMSNs, FITC-labeled EMSNs@CaP, DiI-labeled EM, and FITC and DiI dual-labeled EsPMs. Scale bar, 10 μm. (**E**) SDS-PAGE analysis of protein marker and I–V (EM, SsPMs, EsPMs, EMSNs@CaP, EMSNs). (**F**) N_2_ adsorption/desorption isotherms, (**G**) Pore size distribution curves and (**H**) SAXD pattern of EMSNs and EMSNs@CaP.

The particle size distribution curves and TEM images of different nanoparticles are shown here: Figure 1A,C. EMSNs showed erythrocyte-like shapes with significant disc structures and bowl-shaped depressions. The surface was rough and some pores were visible, and the particle size was about 138 nm. Compared with EMSNs, the surface of EMSNs@CaP was smoother and the particle size was about 154 nm. These results showed that CaP was uniformly deposited on EMSNs. There was a bilayer membrane on the surface of EsPMs and the film thickness was about 10 nm. As a control group, the TEM images of SsPMs exhibited spherical cores and CaP and erythrocyte dual membranes. Meanwhile, the surface charge of bare EMSNs was −29.76 mV due to the large number of silanol on the silica surface. After coating with a CaP membrane, the surface charge of EMSNs@CaP changed from negative to positive and the potential was 1.90 mV. The potential of EsPMs decreased to −9.68 mV since the coating of the erythrocyte membrane obscured the CaP membrane to reduce the exposure of positive charges and the erythrocyte membrane was negatively charged. The reversal of the potential also confirmed the complete fabrication of biomimetic nano-erythrocytes.

The CLSM images and protein bands of SDS-PAGE further proved that the EMs completed coating on the surface of EMSNs@CaP. The FITC-labeled EMSNs@CaP and DiI-labeled EM showed green and red fluorescence, respectively. The EsPMs with two kinds of fluorescence labeling displayed yellow fluorescence, indicating that the EM had been successfully coated on the surface of EMSNs@CaP (Figure 1D). The surface membrane proteins’ distribution of nanoparticles is shown in Figure 1E. The main protein bands of SsPMs (II) and EsPMs (III) were similar to that of EM (I), and there were no protein components on the surface of EMSNs@CaP (IV) and EMSNs (V) without EM coatings (Figure 1E). These results also suggested that membrane proteins were not lost and the EM was successfully coated on the surface of EsPMs, so that EsPMs were able to escape from being eliminated in blood circulation due to comprehensive EM camouflage.

N_2_ adsorption/desorption and the small-angle X-ray diffraction (SAXD) were used to describe the mesoporous structure and calculate the relevant parameters of nanoparticles. The N_2_ adsorption/desorption isotherms and pore size distribution curves of EMSNs and EMSNs@CaP are shown in Figure 1F and Figure 1G, respectively. The BET surface area, pore volume and pore diameter of EMSNs and EMSNs@CaP are shown in Table 1. The apparent hysteresis loop (typical IV isotherm) of the cores could be noticed, indicating that EMSNs and EMSN@CaP existed in the mesoporous structure (Figure 1F), which was consistent with other mesoporous structures reported in the literature [36]. The specific surface area of EMSNs and EMSNs@CaP was 415.43 m^2^/g and 31.95 m^2^/g, calculated by the Brunauer-Emmett-Teller (BET) method. The pore diameter and pore volume of EMSNs were 2.21 nm and 0.64 cm^3^/g, showing that nano-erythrocytes had a favorable drug storage capacity. In addition, it was also proved that EMSNs belong to mesoporous structures according to the pore size (>2 nm), and the pore size distribution curve was similar to mesoporous materials in other studies [37]. Compared with EMSNs, the nitrogen adsorption capacity, pore diameter and pore volume of EMSNs@CaP decreased significantly (Figure 1F,G). This was due to the presence of the CaP membrane that prevented some nitrogen from entering the channels of EMSNs@CaP. These results proved that CaP was successfully coated on the surface of EMSNs. Furthermore, as declared in the SAXD pattern (Figure 1H), EMSNs presented a maximum peak at 2.21°(2θ), demonstrating that EMSNs possessed ordered mesoporous channels. On the contrary, the scattering peak of EMSNs@CaP was not obvious, suggesting that the pore channel presented a disordered distribution.

### 3.2. The In Vitro pH-Responsive Ca^2+^-Release Study

**Figure 2:**
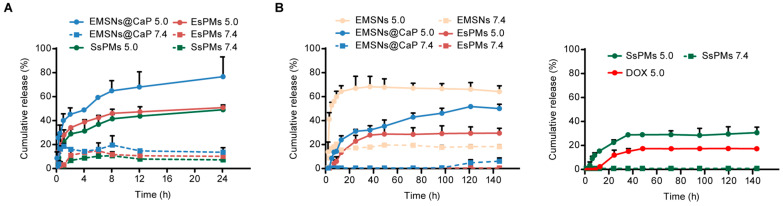
In vitro Ca^2+^ and DOX release of nanoparticles under different pH conditions. (**A**) The Ca^2+^ cumulative release curves of nanoparticles coated with CaP membrane. (**B**) The DOX cumulative release curves of free DOX and DOX-loaded nanoparticles.

The in vitro cumulative release curves of Ca^2+^ at different pH conditions are shown in Figure 2A. At pH 7.4, the maximum Ca^2+^ cumulative release of EMSNs@CaP was only 19.61% (11.77 μg/mL) during the whole experiment. However, at pH 5.0, EMSNs@CaP released Ca^2+^ explosively at the first 1 h interval and the maximum Ca^2+^ cumulative release reached 76.72% (46.03 μg/mL), which showed that the CaP membrane on EMSNs@CaP was sensitive to pH conditions and decomposed to produce a large amount of Ca^2+^. And, Ca^2+^ could affect intracellular ion homeostasis, which led to the generation of ROS, laying a foundation for further research in cell experiments.

After being coated with EM, the Ca^2+^ release of EsPMs and SsPMs significantly decreased both at pH 5.0 and pH 7.4, which proved that coating with EM provided certain protection for the internal structure. This was attributed to the fact that EM reduced the direct contact between the calcium phosphate and the dissolution medium, so the process of degradation slowed down. In addition, the Ca^2+^ cumulative release of EsPMs and SsPMs was 50.89% and 49.15% (30.53 and 29.49 μg/mL) at pH 5.0, respectively, which was obviously higher than that at PH 7.4. This showed that EsPMs and SsPMs still had pH sensitivity after coating EMs.

### 3.3. Drug Loading and In Vitro pH-Responsive Release Studies

The drug loading of DOX-loaded nanoparticles is shown in Table 2. DOX/EMSNs showed a superior drug-loading capacity (30.31%), which was mainly attributed to the large specific surface area and excellent pore structure of EMSNs. After being coated with CaP, some of the drug leaked from the pores during the stirring process, and the drug loading of DOX/EMSNs@CaP was only 28.08%. The drug loading of DOX/EsPMs and DOX/SsPMs was 27.89% and 27.62%, respectively, indicating that coating with EM had little effect on the drug loading.

As shown in Figure 2B, the release of free DOX was about 18.54% (1.11 μg/mL) at pH 5.0 and there was no release at pH 7.4 (it could not be detected). DOX/EMSNs showed relatively slow and little release (about 20%, 1.2 μg/mL, 1.08-fold that of pure DOX) at pH 7.4 due to the electrostatic attraction and hydrogen bonding interaction between DOX and EMSNs. In contrast, at pH 5.0, the DOX release increased significantly and the maximum cumulative release reached 67.96% (4.10 μg/mL, 3.69-fold that of pure DOX) for the decreasing density of hydrogen bonds under an acidic environment. For the CaP membrane as a pH-triggered gatekeeper, DOX in DOX/EMSNs@CaP was released slowly and continuously with the occurrence of degradation. On the one hand, the cumulative drug release was only 30.72% (1.84 μg/mL, 1.66-fold of pure DOX) at 24 h at pH 5.0, which significantly reduced the effect of drug burst release. On the other hand, with the gradual degradation of the CaP membrane, the release of DOX from the pores also accelerated under the acidic environment and the cumulative drug release reached 51.36% (3.08 μg/mL, 2.77-fold that of pure DOX) at 140 h. However, the released amount of DOX/EMSNs@CaP sharply decreased to only 5.71% (0.34 μg/mL, 0.31-fold that of pure DOX) at pH 7.4, proving that limited DOX had a low toxicity for normal tissues and cells. After coating with CaP and erythrocyte dual membranes, the pH-dependent release characterization of DOX was still retained in the drug-delivery system. The DOX’s cumulative release of DOX/EsPMs and DOX/SsPMs was 28.34% and 27.55% (1.70 and 1.65 μg/mL, 1.53- and 1.49-fold that of pure DOX) at pH 5.0, suggesting that the existence of the erythrocyte and CaP membrane delayed the release rate of the drug and the burst release was negligible. At pH 7.4, both DOX/EsPMs and DOX/SsPMs achieved almost no release, which meant that the drug-delivery systems coated with dual membranes were expected to minimize the side effects of DOX and show pH-responsive releases.

### 3.4. Biocompatibility and Degradation Evaluations

**Figure 3:**
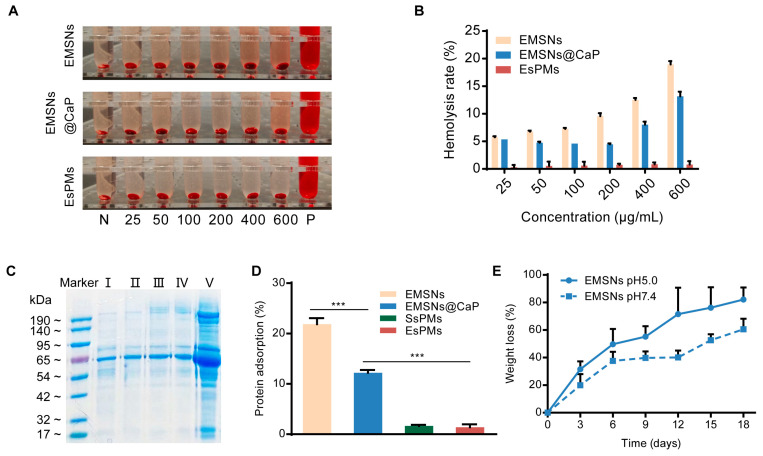
In vitro biocompatibility and degradation studies. (**A**) Hemolysis photographs of nanoparticles with different concentrations (25~600 μg/mL). N, negative group; P, positive group. (**B**) The hemolysis rate of nanoparticles. (**C**) The SDS-PAGE protein analysis of nanoparticles after incubation with plasma. I–Ⅴ: EMSNs, EMSNs@CaP, EsPMs, SsPMs, and plasma. (**D**) The non-specific protein adsorption of nanoparticles after incubation with plasma. Each datum represents the mean ± SD (*n* = 3). *** *p* < 0.001. (**E**) The weight loss profiles of EMSNs at pH 5.0 and pH 7.4.

The hemolysis rate and plasma protein absorption value of nanoparticles are essential indexes to evaluate the quality of intravenous formulations. It was well known that the biocompatibility of erythrocytes was ascendant, so that EsPMs should also possess a low hemolysis rate and protein absorption value. The hemolysis phenomenon and hemolysis rate of EMSNs, EMSNs@CaP and EsPMs are shown in Figure 3A,B. The hemolysis rate of EMSNs and EMSNs@CaP showed a significant concentration dependence in the range (25~600 μg/mL). The supernatant color of the EMSNs changed from white to pink gradually and the maximum hemolysis rate was 18.94%. This was due to the electrostatic interaction between the silanol groups on the surface of EMSNs and membrane proteins, and the high affinity between EMSNs and quaternary ammonium ions in EMs, resulting in its rupture. After being coated with a CaP membrane, the hemolysis rate of EMSNs@CaP decreased significantly due to the shielding of silanol groups, but it still caused hemolysis and did not meet the requirements of intravenous injection. It was worth noting that EsPMs exhibited an extremely low hemolysis rate at 600 µg/mL (0.80%) with even the colorless supernatant. In summary, the EsPMs possessed outstanding blood compatibility due to the coating with the EM and CaP membrane.

The strong adsorption of plasma proteins for nanoparticles increased the possibility of their recognition and clearance by the immune system, further causing a low antitumor effect. Herein, the non-specific protein adsorption of EMSNs, EMSNs@CaP, SsPMs and EsPMs were determined by SDS-PAGE analysis (Figure 3C) and the BCA method (Figure 3D). As expected, the protein band of EsPMs (III) was shallowest in comparison with EMSNs (I), EMSNs@CaP (II), and SsPMs (IV), reflecting that the nanoparticles coated with EM decreased the protein adsorption rate and had a certain immune escape ability. The plasma protein adsorption rate of EMSNs, EMSNs@CaP, and EsPMs were 23.71%, 12.23%, and 1.44%, respectively. As a control group, SsPMs also displayed a low adsorption rate (1.65%), declaring that core shape might not be the vital factor affecting protein adsorption. These results showed that nanoparticles coated with EMs could effectively reduce the plasma protein adsorption rate. Therefore, they were probably not easy to be recognized and cleared by the mononuclear phagocytosis system, thus giving the nanoparticles a longer circulation time in vivo.

The degradation of nanoparticles was an important property for injection administration. As shown in Figure 3E, EMSNs exhibited time-dependent degradation behavior, and the degradation rate of EMSNs at pH 5.0 (82.11%) was higher than that at pH 7.4 (60.51%). These results were conducive to the release of drugs from EMSNs to increase drug efficacy in the tumor microenvironment and reduced the possibility of EMSN accumulation in the body for improving the biosafety of the carrier.

### 3.5. In Vitro Diffusion and Penetration Ability Studies

The primary obstacle of chemotherapy on malignancy breast cancer is that the extracellular matrix (ECM) and high interstitial pressure prevents drugs from penetrating. Some nano-discs have been reported to provide superior mucosal permeability with rotation motions [38]. Therefore, it was supposed that that nano-erythrocytes with erythrocyte-like shapes and biological membranes would probably accomplish an astonishing penetration depth.

**Figure 4:**
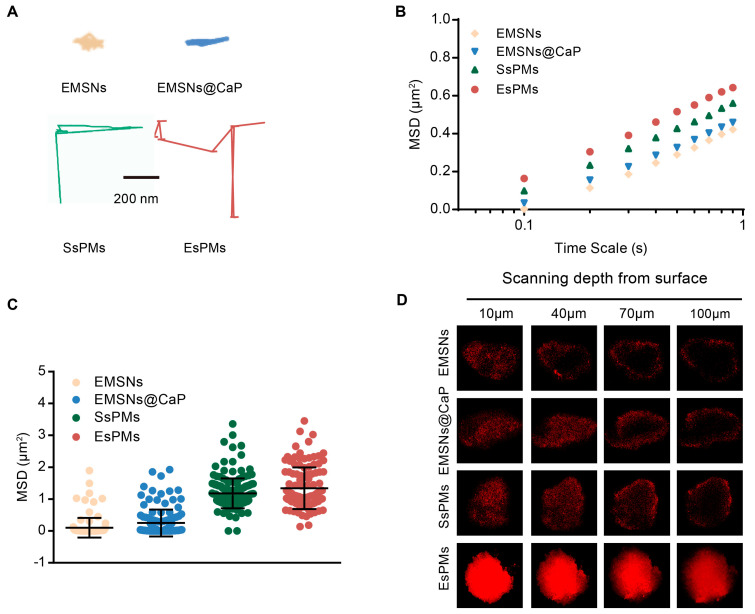
In vitro diffusion and penetration ability studies of nanoparticles. (**A**) The representative trajectories of nanoparticles on a time scale of 1 s. (**B**) The ensemble-averaged geometric MSD as a function of time scale. (**C**) The typical MSD values of nanoparticles at 1 s. (**D**) The penetration of EMSNs, EMSNs@CaP, SsPMs, and EsPMs into the tumor sphere.

As shown in Figure 4A, the representative trajectories for nanoparticles on a time scale of 1 s were recorded. EsPMs displayed the highest penetration ability among these nanoparticles, suggesting that coating with EM could push the core to penetrate into the tumor tissue by the “similar phase dissolution” principle. The order of MSD values was as follows: EsPMs (0.64 μm^2^) > SsPMs (0.56 μm^2^) > EMSNs@CaP ≈ EMSNs (0.42 μm^2^) (Figure 4B,C), which was consistent with the result in Figure 4A. The Deff values of EsPMs were 1.52-, 1.42-, and 1.13-fold higher than EMSNs, EMSNs@CaP and SsPMs, respectively. These results indicated that EsPMs with advantages of both erythrocyte-like shape and EM had excellent diffusion and penetration abilities in simulated tumor stroma.

The results of the tumor sphere penetration experiments of several nanoparticles are shown in Figure 4D. EMSNs and EMSN@CaP groups exhibited less red fluorescence, indicating that the cross-linked ECM prevented the diffusion of nanoparticles, and EMSNs and EMSNs@CaP could only be on the surface of the tumor sphere and could not enter the interior. After coating with EMs, EsPMs showed stronger fluorescence intensity than that of SsPMs at all scan depths, indicating that erythrocyte-like mesoporous silica has a better tumor-penetration ability than spherical mesoporous silica.

### 3.6. Cell Viability Study

**Figure 5:**
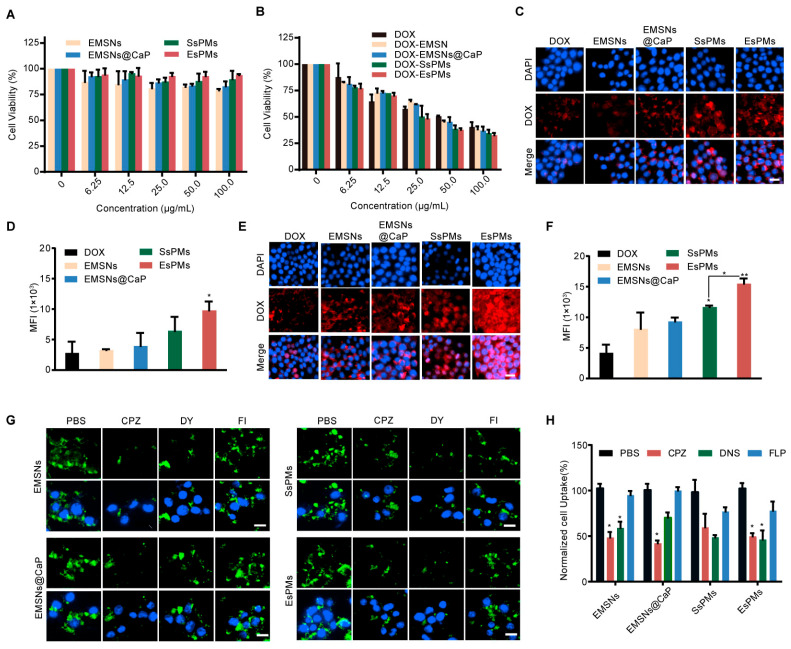
The cell cytotoxicity and cellular uptake studies. In vitro cell viability after incubated with (**A**) blank nanocarriers and (**B**) free DOX and DOX-loaded nanoparticles. The CLSM images of 4T1 cells incubated with free DOX and DOX-loaded nanoparticles for 4 h (**C**) and 8 h (**E**). The mean fluorescence intensity of the 4T1 cells incubated with free DOX and DOX-loaded nanoparticles for 4 h (**D**) and 8 h (**F**). (**G**) The CLSM images of the cellular uptake of nanoparticles in 4T1 cells pretreated with CPZ, DNS, and FLP. (**H**) The qualification of internalized nanoparticles. Scale bar of CLSM images, 20 μm. Each datum represents the mean ± SD (*n* = 3). * *p* < 0.05, ** *p* < 0.01.

An MTT assay was conducted to investigate the in vitro cell cytotoxicity of blank nanocarriers and DOX-loaded nanoparticles (DOX/EMSNs, DOX/EMSNs@CaP, DOX/SsPMs and DOX/EsPMs). As shown in Figure 5A, blank nanocarriers displayed excellent biosafety. The cell viability of SsPMs and EsPMs were above 90% at a concentration range of 0~100 μg/mL. The cytotoxicity of EMSNs and EMSNs@CaP was a little high but still in the safety field. Therefore, all of the results above demonstrated that coating with EM improved the biocompatibility of cores significantly. After loading the drug, all DOX-loaded nanoparticles presented an obviously dose-dependent cell cytotoxicity (Figure 5B). After incubation with 4T1 cells for 24 h, DOX/EMSNs and DOX/EMSNs@CaP groups showed a higher cell cytotoxicity than the free DOX group. The cell viability of DOX/EMSNs and DOX/EMSNs@CaP decreased to 38.28% and 36.99% at the maximum concentration, which was likely related to the enhanced absorption of the erythrocyte-like shape and the generation of ROS induced by the Ca^2+^ produced after the degradation of the CaP membrane. After DOX/EsPMs were co-incubated with 4T1 cells for 24 h, the cell viability (32.67%) was lower than other groups due to the enhanced internalization caused by the EM and the shape effect. In particular, the cytotoxicity of DOX/EsPMs was higher than that of DOX/SsPMs, indicating that DOX-loaded nano-erythrocytes showed better antitumor effects than spherical nanoparticles.

### 3.7. Cellular Uptake and Detailed Mechanism Studies

Confocal laser scanning microscopy (CLSM) was employed to observe the cellular uptake of free DOX and DOX-loaded nanoparticles. The CLSM images are shown in Figure 5C,E; DOX-loaded nanoparticles displayed a time-dependent cellular uptake and exhibited larger red fluorescence areas compared with the free DOX group. The DOX/EMSNs group increased the cellular uptake by 4T1 cells due to their unique erythrocyte-like shape. Moreover, besides the shape effect, the DOX/EMSNs@CaP group consumed H+ in lysosomes due to the decomposition of the CaP membrane at low pH, resulting in the destruction of the lysosome and the release of its captured drug, which promoted DOX release from the pores and further enhanced their fluorescence signals. DOX/EsPMs avoided the phagocytosis of the reticuloendothelial system due to the unique immune escape ability of the EM. Additionally, the red fluorescence of DOX/EsPMs was significantly stronger than that of DOX/SsPMs, indicating that nano-erythrocytes had stronger cell internalization ability than spherical nanoparticles. Therefore, DOX/EsPMs concentrated the advantages of the shape and dual membrane, which enabled more DOX to be released from nanoparticles, resulting in an enhanced red fluorescence signal. The quantitative analysis of cellular uptake (Figure 5D,F) was consistent with CLSM images. After incubation with 4T1 cells for 4 h, the mean fluorescence intensity of DOX/EsPMs group was higher than that of other groups. After incubating for 8 h, the quantitative fluorescence intensity of DOX/EsPMs was 3.6- and 1.3-fold higher than DOX and DOX/SsPMs, respectively. The results showed that the high cellular uptake efficiency of the DOX/EsPMs group was mainly caused by the unique immune escape ability and enhanced cellular internalization of the EM and the erythrocyte-like shape effect.

We next investigated the endocytic mechanism of nanoparticles, which could be a primary basis for further research. Three endocytic inhibitors were applied to investigate the cellular uptake mechanism. Chlorpromazine (CPZ) prevents the clathrin-mediated route [39], filipin (FLP) blocks the caveolin-mediated pathway [40], and dynasore (DNS) is an inhibitor of the both clathrin and caveolin-mediated endocytosis [41]. The CLSM images for the endocytosis of nanoparticles are shown in Figure 5G. We could observe that the cellular uptake of the EMSN and EMSNs@CaP groups decreased significantly when incubated with CPZ while they did not with FLP, suggesting that EMSNs and EMSNs@CaP were endocytosed by the clathrin-mediated route. Additionally, it has been reported that clathrin-mediated endocytosis possesses Ca^2+^ concentration dependence [42]. Therefore, the CaP membrane is decomposed to produce Ca^2+^ in the TME, which can promote clathrin-induced endocytosis, and then increase cellular uptake efficiency. After incubation with DNS, CPZ, and FLP, the cellular uptake of both the EsPMs and SsPMs groups decreased, indicating that EsPMs and SsPMs entered the cell through the clathrin and caveolin-mediated endocytosis pathway. But, after DNS and CPZ treatment, the cellular uptake was significantly lower than that of FLP treatment, indicating that EsPMs and SsPMs depended mainly on the clathrin-mediated endocytosis pathway. The quantitative results of internalized nanoparticles also confirmed the above conclusion (Figure 5H).

### 3.8. Mitochondrial Co-Localization

**Figure 6:**
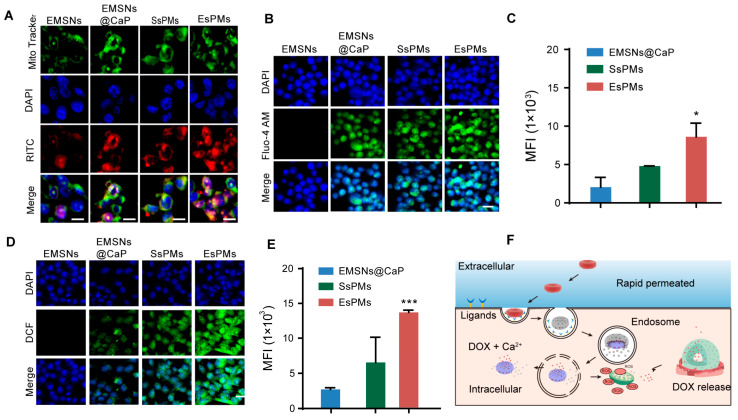
Mitochondrial co-localization, intracellular Ca^2+^ release, ROS detection and antitumor mechanism studies. (**A**) The mitochondrial co-localization of nanoparticles (EMSNs, EMSNs@CaP, EsPMs and SsPMs) in 4T1cells. The CLSM images of intracellular Ca^2+^ release. (**B**,**D**) ROS detection. Scale bar of CLSM images, 20 μm. The mean fluorescence intensity analysis of intracellular Ca^2+^ release (**C**,**E**) and intracellular ROS production. Each datum represents the mean ± SD (*n* = 3). * *p* < 0.05, *** *p* < 0.001. (**F**) The scheme illustration of antitumor mechanism.

Mitochondria are the main source of intracellular ROS, but is easily damaged by excess ROS, which induces cell death and even organ damage [43]. The CLSM was used to demonstrate the mitochondrial co-localization of nanoparticles and the results are shown in Figure 6A. It could be seen that red fluorescence of SsPMs and EsPMs was stronger than that of other groups without the EM, suggesting that SsPMs and EsPMs had enhanced cell internalization abilities due to the coating of EM. Especially, due to the erythrocyte-like shape effect, the red fluorescence of EsPMs was stronger than SsPMs, demonstrating that EsPMs have better cell internalization ability, which was consistent with the results of a cell uptake experiment. In addition, it can be seen that EsPMs have a good co-localization effect on the mitochondrial matrix, showing stronger green fluorescence than other groups. The effective accumulation of EsPMs in mitochondria contributed to the degradation of CaP to produce Ca^2+^ in mitochondria, then a large amount of Ca^2+^ induced the generation of excessive ROS to damage mitochondria, thereby killing cells.

### 3.9. Intracellular Ca^2+^ Release and ROS Detection

The Ca^2+^ release condition of each group is shown in Figure 6B,C. It could be seen that nanoparticles coated with CaP membrane all generated manifest green fluorescence. Among these, the fluorescence intensity of the EsPMs group was significantly higher than that of EMSNs@CaP and SsPMs groups. The results showed that coating with EMs and the erythrocyte-like shape enhanced cell internalization and gathered the EsPMs at tumor sites. The CaP membrane was rapidly degraded in response to the tumor microenvironment, which promoted the production of Ca^2+^.

The CLSM images and quantitative data both exhibited that the production amount of ROS could be ordered as EsPMs > SsPMs >EMSNs@CaP (Figure 6D,E). EMSNs, as a control group, could not provide ROS without a CaP membrane. With the advantages of erythrocyte-like shapes and dual membranes, the fluorescence intensity of EsPMs was higher than that of EMSNs@CaP and SsPMs groups. The processes of EsPMs for Ca^2+^ release and the apoptosis mechanism of tumor cells were described as follows: (1) they rapidly penetrated through the ECM and endocytosed into tumor cells; (2) the CaP membrane was degraded under the acidic tumor microenvironment to produce Ca^2+^, which induced the generation of ROS, and then DOX was released from the pores of nano-erythrocytes; (3) and the oxidative stress appeared to kill tumor cells due to a disturbance in both calcium and ROS homeostasis (Figure 6F).

### 3.10. The In Vivo Circulation Performance

**Figure 7:**
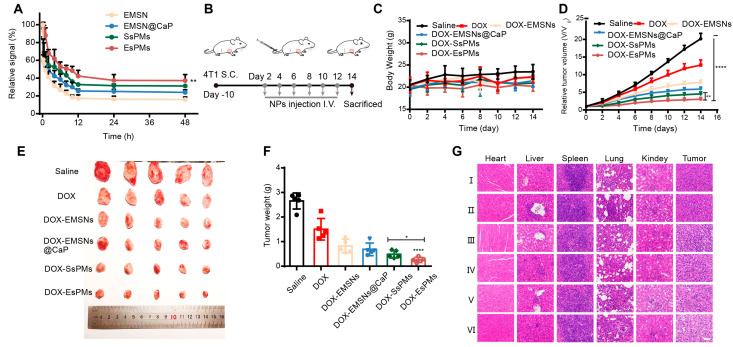
In vivo circulation, antitumor effect and biosafety studies. (**A**) Relative fluorescence intensities in blood samples at different time points. (**B**) The schematic illustration of mice administration. The body weight (**C**) and tumor volume changes (**D**) in 4T1 tumor-bearing mice treated with Saline, DOX, and DOX-loaded nanoparticles in 14 days. (**E**) The tumor images of different groups after the 14 day experiment (*n* = 5). (**F**) The tumor weight of 4T1 tumor-bearing mice treated with Saline, DOX, and DOX-loaded nanoparticles for 14 days. (**G**) H and E staining photographs of tumors and main organs from different groups (I: Saline, II: DOX, III: DOX/EMSNs, IV: DOX/EMSNs@CaP, V: DOX/SsPMs, VI: DOX/EsPMs). Scale bar, 50 μm. Each datum represents the mean ± SD (*n* = 5). * *p* < 0.05, ** *p* < 0.01, **** *p* < 0.0001.

The blood circulation capacity of the nanoparticles (EMSNs, EMSNs@CaP, EsPMs and SsPMs) in vivo was detected by measuring the relative fluorescence intensities of blood samples acquired at predetermined time intervals after injecting FITC-labeled nanocarriers (Figure 7A). After 48 h, the relative signal of the EsPMs group reached 37.19%, which was 2.33- and 1.55-fold higher than that of the EMSN and EMSNs@CaP groups, respectively, indicating that coating with EM could effectively prolong the blood circulation time. For SsPMs, the fluorescence intensity was lower than that of the EsPMs group, suggesting that the spherical nanocarriers had weaker in vivo circulation ability than erythrocyte-like nanocarriers.

### 3.11. The In Vivo Antitumor Effect and Biosafety

The in vivo pharmacodynamics and biosafety experiments were applied to evaluate the antitumor effect and biosafety of nanoparticles on tumor-bearing mice. The administration scheme is shown in Figure 7B. There was no obvious body weight fluctuation for all the treatment groups except for the DOX group (Figure 7C), indicating that the synthesized drug-delivery systems had certain biosafety. The relative tumor volume, collected tumor tissue, and tumor weight were calculated, photographed and recorded, respectively (Figure 7D–F). As shown in Figure 7D, the DOX group showed a certain antitumor effect in the early stage, but with the extension of administration time, the relative tumor volume increased to 12.70 in the later stage and the tumor inhibition effect was not satisfying. The relative tumor volume of DOX/EMSNs and DOX/EMSNs@CaP groups increased slowly, showing a better tumor-inhibition effect. In particular, the relative tumor volume of the DOX/EsPMs group was significantly lower than that of other groups, which was due to the fact that DOX/EsPMs was coated with the EM and CaP dual membranes to obtain a biocompatible surface to “hide” itself, and possessed the superior erythrocyte-like shape to reach a deeper penetration. The images and weights of the tumor in each group are shown in Figure 7E,F. The results showed that the tumor weight of the DOX/EsPMs group was the smallest (0.256 g) and its tumor inhibition reached 90.35%, demonstrating that the treatment effect was the best. Upon reaching the tumor sites, the CaP membrane inside the DOX/EsPMs degraded in the TME, resulting in a large amount of Ca^2+^ poured into the mitochondria to induce excessive ROS generation. At the same time, DOX/EsPMs released a large amount of loaded DOX due to the opening of the pores after the degradation of CaP, achieving dual tumor-cell-killing effects.

Additionally, the histopathological analysis of heart, liver, spleen, lung, kidney and tumor slices was assessed by H and E staining (Figure 7G). The damage of myocardial cells could be seen obviously in the DOX group, which was coherent with the common side effect of DOX, which could produce lipid peroxidation in heart tissue to damage myocardial cells [44]. The myocardial cells of the DOX/EsPMs group were the most complete and without obvious damage. This was mainly because the erythrocyte-like nanoparticles coated with EMs and CaP dual membranes had low toxicity for normal tissues and cells. Moreover, DOX/EsPMs achieved the pH-trigger of drug release in the tumor microenvironment, but achieved zero drug release in the normal physiological tissue environment, reducing the heart damage. In addition, there was no obvious damage in other main organ tissues in preparation groups. For tumor issues, after treatment with DOX and DOX-loaded nanoparticles, tumor cells showed different damage degrees, among which the nuclear atrophy and necrosis of tumor tissue in the DOX/EsPMs group was the most serious, indicating that pH-sensitive DOX/EsPMs based on structure and function bionics could effectively inhibit tumor cell growth and kill tumor cells, and had an expected antitumor effect in vivo.

## 4. Conclusions

In brief, multifunctional biomimetic nano-erythrocytes (EsPMs) were constructed for overcoming a series of barriers in the TME and enhancing antitumor effects. In this study, the erythrocyte-like cores (EMSNs) displayed desirable diffusion and penetration distance in simulated ECM solution. After coating with CaP, EMSNs@CaP exhibited an obviously pH-responsive drug release behavior. In addition, it also has been verified by cell experiments that the Ca^2+^ of CaP degradation induced the production of reactive oxygen species (ROS), and then promoted tumor cell apoptosis. After coating with EM, EsPMs possessed immune escape capacity, enhanced cellular internalization and longer blood circulation time. The in vitro and in vivo experiments indicated a deeper tumor penetration distance (about 100 μm), reliable biocompatibility and high tumor inhibition (90.35%) of EsPMs. In summary, this biomimetic nano-delivery system with advantages of pH-responsive drug release and oxidative stress achieved the dual killing of tumor cells, expanding the novel bionic sight of structure and function to develop a multifunctional nanoplatform for improving antitumor therapy.

## Figures and Tables

**Table 1:** BET surface area, pore volume and pore diameter of EMSNs and EMSNs@CaP.

Samples	S_BET_ (m^2^/g)	W_BJH_ (nm)	V_t_ (cm^3^/g)
EMSNs	415.43	2.21	0.64
EMSNs@CaP	31.95	2.07	0.17

**Table 2:** Drug-loading capacity of carriers.

Drug-Loaded Samples	Drug-Loading Efficiency (%)
DOX/EMSNs	30.31
DOX/EMSNs@CaP	28.08
DOX/EsPMs	27.89
DOX/SsPMs	27.62

## Data Availability

Data are contained within the article.

## References

[1] Sanhai W.R., Sakamoto J.H., Canady R., Ferrari M. (2008). Seven challenges for nanomedicine. Nat. Nanotechnol..

[2] Prabhakar U., Maeda H., Jain R.K., Sevick-Muraca E.M., Zamboni W., Farokhzad O.C., Barry S.T., Gabizon A., Grodzinski P., Blakey D.C. (2013). Challenges and Key Considerations of the Enhanced Permeability and Retention Effect for Nanomedicine Drug Delivery in Oncology. Cancer Res..

[3] Champion J.A., Katare Y.K., Mitragotri S. (2007). Particle shape: A new design parameter for micro- and nanoscale drug delivery carriers. J. Control. Release.

[4] Lee B.J., Cheema Y., Bader S., Duncan G.A. (2021). Shaping nanoparticle diffusion through biological barriers to drug delivery. JCIS Open.

[5] Ahmed H., Gomte S.S., Prathyusha E., Prabakaran A., Agrawal M., Alexander A. (2022). Biomedical applications of mesoporous silica nanoparticles as a drug delivery carrier. J. Drug Deliv. Sci. Technol..

[6] Alyassin Y., Sayed E.G., Mehta P., Ruparelia K., Arshad M.S., Rasekh M., Shepherd J., Kucuk I., Wilson P.B., Singh N. (2020). Application of mesoporous silica nanoparticles as drug delivery carriers for chemotherapeutic agents. Drug Discov. Today.

[7] Gao Y., Sun R., Yu L., Wang W. (2023). Facile preparation of hierarchical mesoporous silica microspheres with tunable porous structure and particle sizes. Ceram. Int..

[8] Lai S.-M., Lai H.-Y., Chou M.-Y. (2014). A facile approach for the tunable wormlike or ordered pore morphology of mesoporous silica: Effect of catalyst types and polyethylene glycol. Microporous Mesoporous Mater..

[9] Liao J., Zhang H., Wang X. (2020). Polydopamine-doped virus-like mesoporous silica coated reduced graphene oxide nanosheets for chemo-photothermal synergetic therapy. J. Biomater. Appl..

[10] Hao N., Nie Y., Zhang J.X.J. (2018). Biomimetic hierarchical walnut kernel-like and erythrocyte-like mesoporous silica nanomaterials: Controllable synthesis and versatile applications. Microporous Mesoporous Mater..

[11] Wang N., Li J., Wang J., Nie D., Jiang X., Zhuo Y., Yu M. (2022). Shape-directed drug release and transport of erythrocyte-like nanodisks augment chemotherapy. J. Control. Release.

[12] Roggers R.A., Joglekar M., Valenstein J.S., Trewyn B.G. (2014). Mimicking Red Blood Cell Lipid Membrane to Enhance the Hemocompatibility of Large-Pore Mesoporous Silica. ACS Appl. Mater. Interfaces.

[13] Yue J., Wang Z., Shao D., Chang Z., Hu R., Li L., Luo S.-Z., Dong W.-F. (2018). Cancer cell membrane-modified biodegradable mesoporous silica nanocarriers for berberine therapy of liver cancer. RSC Adv..

[14] Nie D., Dai Z., Li J., Yang Y., Xi Z., Wang J., Zhang W., Qian K., Guo S., Zhu C. (2020). Cancer-Cell-Membrane-Coated Nanoparticles with a Yolk-Shell Structure Augment Cancer Chemotherapy. Nano Lett..

[15] Liu C.-M., Chen G.-B., Chen H.-H., Zhang J.-B., Li H.-Z., Sheng M.-X., Weng W.-B., Guo S.-M. (2019). Cancer cell membrane-cloaked mesoporous silica nanoparticles with a pH-sensitive gatekeeper for cancer treatment. Colloids Surf. B Biointerfaces.

[16] Hu Y., Ke L., Chen H., Zhuo M., Yang X., Zhao D., Zeng S., Xiao X. (2017). Natural material-decorated mesoporous silica nanoparticle container for multifunctional membrane-controlled targeted drug delivery. Int. J. Nanomed..

[17] Lodoso-Torrecilla I., van den Beucken J.J.J.P., Jansen J.A. (2021). Calcium phosphate cements: Optimization toward biodegradability. Acta Biomater..

[18] Tang Z., Zhou Y., Sun H., Li D., Zhou S. (2014). Biodegradable magnetic calcium phosphate nanoformulation for cancer therapy. Eur. J. Pharm. Biopharm..

[19] Li S., Zhang L., Zhang H., Mu Z., Li L., Wang C. (2017). Rationally Designed Calcium Phosphate/Small Gold Nanorod Assemblies Using Poly(acrylic acid calcium salt) Nanospheres as Templates for Chemo-photothermal Combined Cancer Therapy. ACS Biomater. Sci. Eng..

[20] Duc-Viet N., Jiang S., He C., Lin Z., Lin N., Anh-Tuan N., Kang L., Han M.-Y., Liu X.-Y. (2016). Elevating Biomedical Performance of ZnO/SiO_2_@Amorphous Calcium Phosphate—Bioinspiration Making Possible the Impossible. Adv. Funct. Mater..

[21] Nomoto T., Fukushima S., Kumagai M., Miyazaki K., Inoue A., Mi P., Maeda Y., Toh K., Matsumoto Y., Morimoto Y. (2016). Calcium phosphate-based organic-inorganic hybrid nanocarriers with pH-responsive on/off switch for photodynamic therapy. Biomater. Sci..

[22] Xu L., Tong G., Song Q., Zhu C., Zhang H., Shi J., Zhang Z. (2018). Enhanced Intracellular Ca^2+^ Nanogenerator for Tumor-Specific Synergistic Therapy via Disruption of Mitochondrial Ca^2+^ Homeostasis and Photothermal Therapy. ACS Nano.

[23] Liu J., Hu X., Jin S., Liang X.-J., Ma X. (2022). Enhanced anti-tumor activity of a drug through pH-triggered release and dual targeting by calcium phosphate-covered mesoporous silica vehicles. J. Mater. Chem. B.

[24] Yang G., Phua S.Z.F., Bindra A.K., Zhao Y. (2019). Degradability and Clearance of Inorganic Nanoparticles for Biomedical Applications. Adv. Mater..

[25] Rao L., Bu L.-L., Xu J.-H., Cai B., Yu G.-T., Yu X., He Z., Huang Q., Li A., Guo S.-S. (2015). Red Blood Cell Membrane as a Biomimetic Nanocoating for Prolonged Circulation Time and Reduced Accelerated Blood Clearance. Small.

[26] Hu C.-M.J., Zhang L., Aryal S., Cheung C., Fang R.H., Zhang L. (2011). Erythrocyte membrane-camouflaged polymeric nanoparticles as a biomimetic delivery platform. Proc. Natl. Acad. Sci. USA.

[27] Schuerch C.M., Forster S., Bruehl F., Yang S.H., Felley-Bosco E., Hewer E. (2018). The “don’t eat me” signal CD47 is a novel diagnostic biomarker and potential therapeutic target for diffuse malignant mesothelioma. Oncoimmunology.

[28] Oldenborg P.A., Zheleznyak A., Fang Y.F., Lagenaur C.F., Gresham H.D., Lindberg F.P. (2000). Role of CD47 as a marker of self on red blood cells. Science.

[29] Li M., Jiang S., Simon J., Passlick D., Frey M.-L., Wagner M., Mailaender V., Crespy D., Landfester K. (2021). Brush Conformation of Polyethylene Glycol Determines the Stealth Effect of Nanocarriers in the Low Protein Adsorption Regime. Nano Lett..

[30] Zhang J., Tang H., Shen Y., Yu Q., Gan Z. (2018). Shell-Sheddable Poly(N-2-hydroxypropyl methacrylamide) Polymeric Micelles for Dual-Sensitive Release of Doxorubicin. Macromol. Rapid Commun..

[31] Maso K., Grigoletto A., Raccagni L., Bellini M., Marigo I., Ingangi V., Suzuku A., Hirai M., Kamiya M., Yashioka H. (2020). Poly(L-glutamic acid)-co-poly(ethylene glycol) block copolymers for protein conjugation. J. Control. Release.

[32] Yang Q., Lai S.K. (2015). Anti-PEG immunity: Emergence, characteristics, and unaddressed questions. Wiley Interdiscip. Rev. Nanomed. Nanobiotechnol..

[33] Wang J., Pan H., Li J., Nie D., Zhuo Y., Lv Y., Wang N., Chen H., Guo S., Gan Y. (2023). Cell membrane-coated mesoporous silica nanorods overcome sequential drug delivery barriers against colorectal cancer. Chin. Chem. Lett..

[34] Dong N., Liu Z., He H., Lu Y., Qi J., Wu W. (2023). “Hook&Loop” multivalent interactions based on disk-shaped nanoparticles strengthen active targeting. J. Control. Release.

[35] Dey K., Agnelli S., Borsani E., Sartore L. (2021). Degradation-Dependent Stress Relaxing Semi-Interpenetrating Networks of Hydroxyethyl Cellulose in Gelatin-PEG Hydrogel with Good Mechanical Stability and Reversibility. Gels.

[36] Varga G., Somosi Z., Kónya Z., Kukovecz A., Pálinkó I., Szilagyi I. (2021). A colloid chemistry route for the preparation of hierarchically ordered mesoporous layered double hydroxides using surfactants as sacrificial templates. J. Colloid Interface Sci..

[37] Muráth S., Varga T., Kukovecz A., Kónya Z., Sipos P., Palinko I., Varga G. (2022). Morphological aspects determine the catalytic activity of porous hydrocalumites: The role of the sacrificial templates. Mater. Today Chem..

[38] Kuai R., Singh P.B., Sun X., Xu C., Najafabadi A.H., Scheetz L., Yuan W., Xu Y., Hong H., Keskin D.B. (2020). Robust Anti-Tumor T Cell Response with Efficient Intratumoral Infiltration by Nanodisc Cancer Immunotherapy. Adv. Ther..

[39] Plaze M., Attali D., Prot M., Petit A.-C., Blatzer M., Vinckier F., Levillayer L., Chiaravalli J., Perin-Dureau F., Cachia A. (2021). Inhibition of the replication of SARS-CoV-2 in human cells by the FDA-approved drug chlorpromazine. Int. J. Antimicrob. Agents.

[40] Orlandi P.A., Fishman P.H. (1998). Filipin-dependent inhibition of cholera toxin: Evidence for toxin internalization and activation through caveolae-like domains. J. Cell Biol..

[41] Abban C.Y., Bradbury N.A., Meneses P.I. (2008). HPV16 and BPV1 infection can be blocked by the dynamin inhibitor dynasore. Am. J. Ther..

[42] Yao L.-H., Rao Y., Varga K., Wang C.-Y., Xiao P., Lindau M., Gong L.-W. (2012). Synaptotagmin 1 Is Necessary for the Ca^2+^ Dependence of Clathrin-Mediated Endocytosis. J. Neurosci..

[43] Ott M., Gogvadze V., Orrenius S., Zhivotovsky B. (2007). Mitochondria, oxidative stress and cell death. Apoptosis.

[44] Hajra S., Patra A.R., Basu A., Bhattacharya S. (2018). Prevention of doxorubicin (DOX)-induced genotoxicity and cardiotoxicity: Effect of plant derived small molecule indole-3-carbinol (I3C) on oxidative stress and inflammation. Biomed. Pharmacother..

